# Deciphering Drug Resistance: Investigating the Emerging Role of Hyaluronan Metabolism and Signaling and Tumor Extracellular Matrix in Cancer Chemotherapy

**DOI:** 10.3390/ijms25147607

**Published:** 2024-07-11

**Authors:** Daiana L. Vitale, Arianna Parnigoni, Manuela Viola, Evgenia Karousou, Ina Sevic, Paola Moretto, Alberto Passi, Laura Alaniz, Davide Vigetti

**Affiliations:** 1 Laboratorio de Microambiente Tumoral, Centro de Investigaciones Básicas y Aplicadas (CIBA), Universidad Nacional del Noroeste de la Provincia de Buenos Aires, Junín B6000, Argentina; dlvitale@comunidad.unnoba.edu.ar (D.L.V.); isevic@comunidad.unnoba.edu.ar (I.S.); ldalaniz@comunidad.unnoba.edu.ar (L.A.); 2 Centro de Investigaciones y Transferencia del Noroeste de la Provincia de Buenos Aires (CITNOBA), UNNOBA-UNSAdA-CONICET, Junín 6000, Argentina; 3 Department of Medical Biochemistry and Microbiology, Uppsala University, SE-751 23 Uppsala, Sweden; arianna.parnigoni@imbim.uu.se; 4 Dipartimento di Medicina e Chirurgia, Universitá degli Studi dell’Insubria, 21100 Varese, Italy; manuela.viola@uninsubria.it (M.V.); jenny.karousou@uninsubria.it (E.K.); paola.moretto@uninsubria.it (P.M.); albeerto.passi@uninsubria.it (A.P.)

**Keywords:** hyaluronan, HAS2, HAS2-AS1, UGDH, chemotherapy, drug resistance, 4-MU

## Abstract

Hyaluronan (HA) has gained significant attention in cancer research for its role in modulating chemoresistance. This review aims to elucidate the mechanisms by which HA contributes to chemoresistance, focusing on its interactions within the tumor microenvironment. HA is abundantly present in the extracellular matrix (ECM) and binds to cell-surface receptors such as CD44 and RHAMM. These interactions activate various signaling pathways, including PI3K/Akt, MAPK, and NF-κB, which are implicated in cell survival, proliferation, and drug resistance. HA also influences the physical properties of the tumor stroma, enhancing its density and reducing drug penetration. Additionally, HA-mediated signaling contributes to the epithelial–mesenchymal transition (EMT), a process associated with increased metastatic potential and resistance to apoptosis. Emerging therapeutic strategies aim to counteract HA-induced chemoresistance by targeting HA synthesis, degradation, metabolism, or its binding to CD44. This review underscores the complexity of HA’s role in chemoresistance and highlights the potential for HA-targeted therapies to improve the efficacy of conventional chemotherapeutics.

## 1. Introduction

Cancer remains one of the most formidable health challenges of the 21st century. Despite significant advances in cancer treatment, drug resistance continues to pose a substantial obstacle to successful therapy. Drug resistance in cancer refers to the ability of cancer cells to withstand the effects of chemotherapy, targeted therapy, and immunotherapy, thus leading to treatment failure and disease progression. This resistance can develop over time as cancer cells mutate or adapt in response to treatment. Cancer cells can develop drug resistance through several mechanisms. These include the accumulation of genetic mutations that make the cells less susceptible to drugs, the activation of drug efflux pumps that reduce the drug concentration inside the cell, the promotion of survival and growth pathways that evade the toxic effects of chemotherapeutics, and the acquisition of a stem cell-like phenotype [1].

A critical factor contributing to this phenomenon is the tumor microenvironment (TME), which plays a pivotal role in mediating drug resistance in different ways [2]. The TME refers to the complex surroundings in which a tumor exists, including various cell types and molecules such as immune cells, blood vessels, fibroblasts, signaling molecules, and the extracellular matrix (ECM). The TME is associated with an altered ECM, with its composition, structure, and quantity varying throughout tumor progression and between different tumor types [3]. The tumor-associated ECM differs from that in a homeostatic state, much more resembling the ECM found in tissue repair and fibrosis processes, capable of generating what we propose to call “stromal resistance” [2]. Within this complex milieu, hyaluronan (HA) has emerged as a key player in modulating chemoresistance. HA is abundantly present in the ECM of many tissues and tumors, where it influences several cellular processes, including proliferation, migration, and survival [4].

The relationship between HA and chemoresistance is multifaceted, involving a dynamic interplay between HA and cellular signaling pathways, receptor interactions, and the modulation of drug delivery and efficacy. HA exerts its effects through interactions with cell-surface receptors such as CD44 and Receptor for Hyaluronan-Mediated Motility (RHAMM), initiating signaling cascades that can lead to altered cell adhesion, migration, and survival, ultimately promoting a chemoresistant phenotype. Moreover, HA-rich ECM can act as a physical barrier to drug penetration, further complicating treatment strategies.

Even though HA is ubiquitously found in every tissue and its structure and function are fundamentally conserved among all vertebrates, HA exerts differential roles in various cell types, even within the TME, depending on the molecules it interacts with (i.e., HA receptors, proteoglycans, fibronectin, collagens, hyaladherins, etc.) [5]. By affecting tumor cell metabolism, immune cell function, fibroblast activity, and angiogenesis, HA creates a multifaceted support system that enables tumors to grow, evade immune detection, and metastasize. Understanding these cell-type-specific interactions provides valuable insights into potential therapeutic targets aimed at disrupting HA-mediated support systems within the TME, offering new avenues for cancer treatment [6,7,8].

This review aims to provide a comprehensive overview of the current understanding of HA’s role in chemoresistance. We will explore the molecular mechanisms underlying HA-mediated chemoresistance, including receptor interactions and downstream signaling pathways, HA metabolism, and its implications in the TME, focusing on the impact on drug delivery and efficacy.

## 2. ECM and HA

The ECM is a complex network of molecules surrounding and supporting cells within tissues and organs in multicellular organisms. It consists of various proteins, such as collagen, elastin, fibronectin, and laminin, as well as polysaccharides like glycosaminoglycans (GAGs) and proteoglycans (PGs) [9,10]. These components are crucial for tissue integrity, mechanical support, and the regulation of cell behavior, influencing processes such as cell adhesion, migration, proliferation, and differentiation.

The ECM can be categorized into the basal membrane and the interstitial matrix, each with distinct components reflecting their specific functions. The basal membrane acts as a scaffold, anchoring epithelial tissues to the underlying connective tissue and serving as a selective barrier for molecules and cells. It orchestrates cell organization and differentiation through interactions with cell-surface receptors and is primarily composed of type IV collagen, laminins, nidogens, perlecan, and PGs. Situated below the basal membrane, the interstitial matrix forms a three-dimensional network of non-cellular materials that envelops and supports cells in most tissues. This matrix functions as a biological adhesive, maintaining tissue integrity, providing structural support, modulating cell communication, and influencing cellular behavior. It is composed of fibrous proteins, predominantly type I and II collagens, fibronectin, elastin, PGs, and GAGs [11].

The ECM is not merely a passive scaffold; it actively participates in tissue development, repair, and homeostasis [12]. Interestingly, it is widely acknowledged that during pathogenesis, such as inflammation or tumorigenesis, the ECM undergoes significant remodeling processes, leading to the concept of a “healthy” ECM versus an “altered or pathological” ECM [13]. PGs and GAGs are among the ECM components that undergo substantial changes during these remodeling processes. GAGs are a family of long, linear polysaccharides (complex carbohydrates) consisting of repeating disaccharide units. These molecules are typically covalently linked to core proteins, forming PGs. Chondroitin sulfate (CS), dermatan sulfate (DS), keratan sulfate (KS), and heparan sulfate (HS) are typical GAGs characterized by a high negative charge due to sulfate or carboxyl groups, which can attract and bind positively charged ions and water molecules. This property contributes to the gel-like consistency of the ECM and provides mechanical properties to tissues. The great variability of GAGs’ chemical modifications (i.e., sulfation, acetylation, and epimerization) and the polysaccharide chain length are finely tuned processes, and their roles in cell biology are still partially understood [13].

**Figure 1:**
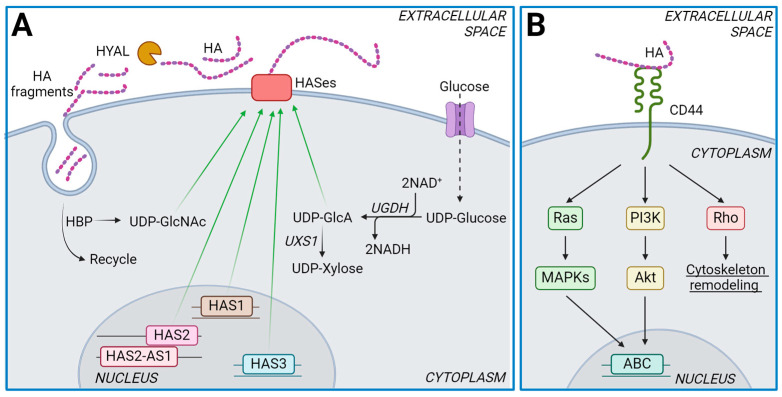
(**A**). Schematic representation of HA synthesis. The figure illustrates the synthesis of UDP-GlcA by the enzyme UGDH, which catalyzes the double oxidation of carbon 6 of glucose to glucuronate, eventually producing 2 NADH molecules. The synthesis of UDP-GlcNAc is achieved through the hexosamine biosynthetic pathway (HBP) or by recycling GlcNAc during HA turnover. The enzyme UXS1 catalyzes the decarboxylation of UDP-GlcA to UDP-Xylose. (**B**). Schematic representation of CD44, the main HA receptor on the cell surface, and the alternative signaling pathways downstream of CD44, including Ras, PI3K, and Rho. These signaling cascades ultimately lead to the activation of ABC transporters in the nucleus.

HA, also known as hyaluronic acid or hyaluronate, is considered a typical GAG due to its unusual characteristics. Unlike most GAGs, HA lacks chemical modifications and is a very large polysaccharide, reaching millions of Daltons (Da), compared to the other GAGs which typically range from several-thousand to several-hundred-thousand Da. The disaccharide unit of HA consists of D-glucuronic acid and N-acetyl-D-glucosamine linked through alternating β-1,3 and β-1,4 glycosidic bonds. Furthermore, unlike most GAGs which are synthesized in the Golgi apparatus, HA is synthesized at the plasma membrane by a family of three isoenzymes known as hyaluronan synthases (HAS1, 2, and 3) (Figure 1A) [5,14]. HASes are highly complex enzymes as they initiate polymer formation from the two UDP-sugar precursors (UDP-glucuronic acid, UDP-GlcA and UDP-N-acetylglucosamine, UDP-GlcNAc) at the cytosolic face, catalyze the formation of the two different types of glycosidic bonds, and then extrude the nascent polymer chain directly into the extracellular space without attachment to a core protein [15].

The turnover of HA is typically rapid, with several degrading enzymes, known as hyaluronidases (HYALs), catalyzing the cleavage of the glycosidic bonds within the HA polymer [16]. These enzymes (HYAL1, HYAL2, HYAL3, PH20, CEMIP, and TMEM2) work together to break down HA chains into smaller fragments, which surprisingly exhibit biological activity often opposite to that of the native high molecular mass molecule (10^7^ Da) [17,18]. Additionally, it is noteworthy that HA can undergo degradation to fragments smaller than 200 Da by reactive oxygen species (ROS) under both physiological and pathological conditions [19]. The activity of these molecules results in changes in the molecular mass of HA, subsequently affecting its function.

High-molecular-mass HA (HMW HA) is ubiquitously distributed in tissues where, thanks to its high hydrophilicity, it contributes to the viscoelastic properties of connective tissues like synovial fluid in joints and vitreous humor in the eye, aiding in lubrication and shock absorption. On the other hand, low-molecular-mass HA (LMW HA) is typically accumulated under pathological conditions, including cancer, fibrosis, and inflammation [6]. Interestingly, HA also interacts with cell-surface receptors such as CD44 and Hyaluronan-mediated motility receptor (RHAMM), influencing cell behavior, migration, and proliferation. Additionally, it participates in wound healing, tissue repair, and regulating inflammation (Figure 1B) [19]. HA also facilitates crosstalk between tumor and stromal cells [20].

HA accumulation in the TME also participates in modulating TME-related immunity. HA-CD44 interaction inhibits the antitumor activity of natural killer and T cells by facilitating macrophage infiltration and differentiation into immunosuppressive tumor-associated macrophages (TAMs). Interestingly, HA and activated M2-like TAMs correlate with higher malignancy in breast cancer, where the HA-CD44 interaction induces TAMs via the HA-CD44-ERK1/2-STAT3 pathway [21]. It has to be highlighted that, depending on its molecular mass, HA’s effects on macrophage activation vary—HMW-HA promotes anti-inflammatory macrophages, whereas LMW HA induces inflammatory macrophages [22]. Additionally, HA-rich TMEs obstruct drug delivery and immune cell infiltration, as observed in pancreatic cancer [23]. Targeted depletion of stromal HA enhances CD8^+^ T cell infiltration and antitumor activity. HA also recruits highly activated regulatory T cells (Tregs), increasing their immunosuppressive activity and impairing immune responses [24].

Overall, HA’s unique structure, synthesis, and biological functions make it a unique GAG, playing a key role in maintaining homeostasis and influencing pathological states [25].

## 3. HA and Its Interaction with the ECM in Cancer

The roles of HA in tumor drug resistance can be categorized as follows: intracellular signaling via its receptors, which modulates cell proliferation and anti-apoptotic pathways; regulation of multidrug resistance (MDR) mechanisms, primarily through ATP-binding cassette (ABC) transporter expression and functionality; control of the differentiation and behavior of cancer stem cells (CSCs); HA’s function as a biophysical barrier, affecting vascularity, angiogenesis, and drug delivery within the tumors; the induction of epigenetic changes. This section will delve into the specific properties of HA concerning the tumor ECM and anticancer therapy, while the interaction with its main receptor CD44 will be explored in Section 4.4.

MDR poses a significant challenge in cancer therapy, as cancer cells develop decreased susceptibility to drugs through several mechanisms [26]. One such mechanism involves increased drug efflux, where drugs are actively expelled from the cell via ABC transporters in the cell membrane [27]. Studies have shown that HA upregulates the expression and functionality of ABC transporters such as ABCB1, ABCC1, BCRP, and ABCG2 [28] (Figure 1B). Since HA function is influenced by its molecular mass, HA fragmentation by HYALs or ROS activity profoundly affects different tumor types. For instance, HA fragments have been found to reduce the expression and function of ABC transporters in lymphoma cell lines, rendering them more sensitive to vincristine [29]. Similarly, in malignant gliomas, HA oligomers inhibit the activation of protein kinase B (PKB, Akt), decrease ABCG2 expression, and enhance methotrexate cytotoxicity [30]. Furthermore, HA fragments can displace intact HA molecules from cell-surface receptors, disrupting HA signaling. Interestingly, vertebrate cells can secrete HA via multidrug transporters [31,32], a mechanism also exploited by tumor cells. Our research has shown that the accumulation of LMW HA in the ECM during doxorubicin treatment alters drug intracellular accumulation and modulates the expression of ABC transporters in a T-cell lymphoma model [33]. Collectively, these findings suggest that the presence of HA in the tumor ECM exacerbates the potential development of chemoresistance.

Angiogenesis is a well-known contributor to chemoresistance. Angiogenesis can lead to increased tumor hypoxia, prompting the production of pro-angiogenic factors that stimulate the growth of abnormal and leaky blood vessels. These vessels make it challenging for anticancer drugs to reach tumor cells. Additionally, angiogenesis can enhance drug efflux by upregulating the expression of drug transporters in endothelial cells, thus reducing the effectiveness of chemotherapy [34]. Our research on HA has also revealed that the accumulation of LMW HA in the ECM modulates the migratory behavior of tumor-associated endothelial cells during doxorubicin treatment in triple-negative breast cancer (TNBC) and osteosarcoma microenvironments. Moreover, murine models of T-cell lymphoma and mammary adenocarcinoma revealed that the accumulation of HA in the TME induced a pro-angiogenic effect on tumors, even during in vivo doxorubicin treatment [33]. This highlights a novel mechanism through which tumor-derived HA in the ECM modulates the response of cancer cells to doxorubicin. Moreover, the TME often becomes hypoxic, prompting tumor cell populations to acquire CSC-like phenotypes. CSCs are a small subpopulation of cells within a tumor possessing stem-cell-like properties, including self-renewal and multilineage differentiation capabilities, and, interestingly, resistance to conventional cancer treatments, mainly due to enhanced DNA repair mechanisms, increased expression of drug efflux pumps, and the ability to remain in a dormant state. CSCs are thus responsible for tumor complexity and diversity, initiation, progression, recurrence, and metastasis [35]. Notably, not all CSCs exhibit the same characteristics, which vary significantly between different types of tumors. Importantly, CD44 has emerged as a CSC marker in several tumor types (e.g., breast, colorectal, lung, and pancreatic cancer) [4,36]. Generally speaking, a high number of CSCs correlates with higher tumor aggressiveness and poor patient prognosis. In a breast carcinoma model, it has been shown that HA regulates the energy metabolism of CSCs under hypoxic conditions. Increased HA accumulation promotes Hypoxia-inducible factor 1-alpha (HIF-1α) signaling by accelerating flux through the hexosamine biosynthetic pathway, the latter being essential for both HA precursors synthesis [37]. This activation of HIF-1α signaling drives a metabolic reprogramming towards glycolysis and the acquisition of CSC-like properties, thereby rendering tumor cells more resistant to chemotherapy.

Recent observations indicate that HA hinders blood vessel function and, in turn, drug delivery, acting as a biophysical barrier. Provenzano et al. documented that HA mediates chemoresistance in pancreatic ductal adenocarcinoma by increasing the interstitial fluid pressure and inducing vascular collapse, eventually creating substantial barriers to the perfusion, diffusion, and convection of small-molecule drugs [23,38]. Additionally, it has been demonstrated that HA can directly bind to doxorubicin, forming strong interactive forces that hinder its cellular entry into tumor tissue, as observed in a breast cancer model [39].

Recently, epigenetics has emerged as a significant factor contributing to chemoresistance. Epigenetic modifications, including DNA methylation, histone modifications, and non-coding RNAs, play a crucial role in promoting uncontrolled cell growth, survival, and chemoresistance. As regards the ECM, the relationship between DNA methylation and HA production in pancreatic ductal adenocarcinoma has been investigated. The findings revealed a remarkable increase in HA production in all pancreatic cancer cell lines treated with a DNA methylation inhibitor or cells where DNA methyltransferase 1 was knocked down. This enhanced HA production coincided with a significant upregulation of HAS2 and HAS3 expression [40]. Furthermore, it has been shown that the interaction between HA and CD44 induces epigenetic alterations mediated by histone methyltransferase DOT1L in head and neck CSCs. This mechanism drives miR-10 expression, promoting Rho GTPase activity and upregulation of survival proteins. This coordinated program favors the acquisition of CSC properties, including enhanced tumor cell invasion and resistance to chemotherapy [41]. This novel insight suggests potential avenues for developing epigenetic-based therapies targeting HA, which could lead to improved treatment outcomes for cancer patients. In this context, we are currently evaluating alterations in HA metabolism and its association with epigenetic and genetic alterations in BRCA1 and BRCA2 DNA repair genes, as potential biomarkers in breast and colon cancer patients (unpublished results). Mutations or epigenetic modifications in these genes can inhibit their function, thus promoting tumor progression [42]. Specifically, BRCA1 methylation inhibited its function in breast cancer, suggesting the clinical use of PARP1 inhibitors or cisplatin in patients with this alteration. This therapeutic approach could effectively induce tumor cell death, providing personalized therapy [43]. Therefore, unraveling the molecular connections between HA and DNA repair pathways holds promise for developing innovative therapeutic strategies to treat cancer.

## 4. Exploring New Roles of HA Metabolism in Cancer Treatment

As previously described elsewhere [4,19], HA metabolism is a highly demanding energy process, even if ATP is not required for HASes activity. Tumor cells undergo a huge metabolic reprogramming, with a switch from an ATP-based metabolism to one focused on maximizing the production of macromolecules, including nucleotides, proteins, lipids, and glycans. As a result, cancer cells produce more carbon skeletons, nitrogen, and NADPH to sustain anaerobic reactions [44]. Interestingly, as an effect of the so-called Warburg effect, glucose is efficiently converted into UDP-glucose through glycogen metabolism, eventually leading to UDP-GlcA by UGDH enzymatic activity. UDP-GlcA is not only essential for GAG synthesis but is also involved in linking GAG to a core protein and is a substrate for detoxification reactions and hormone turnover. Similarly, UDP-GlcNAc is also present with high levels in tumors, where it is involved in GAG synthesis and protein O-GlcNAcylation. Notably, O-GlcNAcylation both stabilizes HAS2 activity and induces the p65 Nf-кB subunit to upregulate HAS2-AS1 transcription, eventually optimizing HA synthesis [45,46]. One last master regulator of HA synthesis is AMPK. Its activation induces HAS2 phosphorylation and decreases HA synthesis. Since the role of AMPK is to maintain an elevated ATP/AMP ratio, its pathway is often downregulated in many tumors [47]. This altered metabolism in tumors leads to an increased HA production which, in turn, binds to CD44, thus inducing downstream signaling pathways, including the PI3K/Akt, HIF1α and AMPK/mTOR, and enhancing glycolysis, mitochondrial biogenesis, and lipid synthesis to support the high metabolic demands of tumor cells [48,49,50,51].

Differences in the levels and properties of HA within the tumor ECM serve as biosensors for chemoresistance. Additionally, it has been demonstrated that increased synthesis of this GAG is not the only factor associated with chemoresistance development. Recent research has also focused on the mechanisms related to HA metabolism and cellular signaling pathways triggered by HA [52]. Strategically targeting HA synthesis, degradation, and signaling pathways, especially when in combination with conventional chemotherapy, holds significant promise for improving the treatment of chemoresistant cancers.

### 4.1. Role of HASes in Chemotherapy

As previously discussed, HA significantly influences cancer, generally promoting tumor progression [53]. Since all three HASes can synthesize HA, each plays a pivotal role in HA-driven tumorigenesis [4]. However, from a pharmacological standpoint, the only available drug to inhibit HA synthesis is 4-methylumbelliferone (4-MU, hymecromone) [54]. 4-MU is a natural organic compound derived from coumarin, specifically 7-hydroxy-4-methylcoumarin, found in various plants. Originally developed in the 1960s for biliary colic, it is currently used to regulate bile flow and treat urinary tract disorders in several countries.

**Figure 2:**
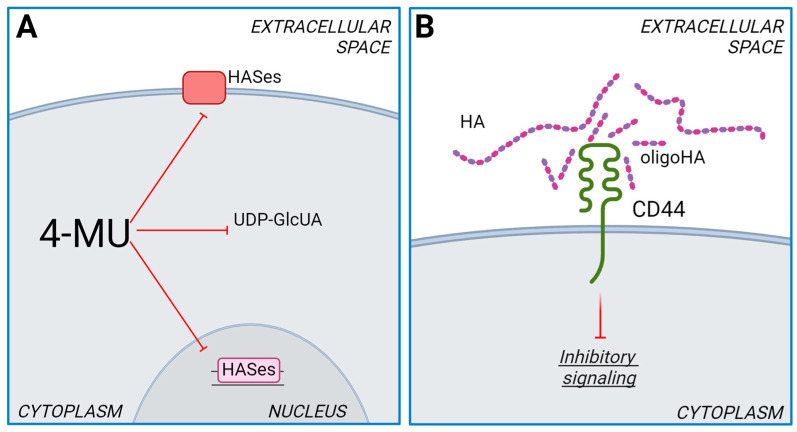
(**A**). Schematic representation of HA synthesis inhibition by 4-MU. 4-MU blocks all HASes by reducing the cytosolic concentration of the precursor UDP-GlcA and inhibits the expression of HASes gene in the nucleus. (**B**). Schematic representation of CD44 inhibition by short HA oligosaccharides while competing with high molecular mass HA for binding to the receptor.

The mechanism of HA synthesis inhibition of 4-MU is complex and can act on multiple levels. 4-MU is converted to 4-methylumbelliferone glucuronide (4-MUG) by UDP-glucuronosyltransferases (UGTs) [55]. These enzymes consume cytosolic UDP-GlcA, a crucial substrate for HA synthesis. Therefore, the reduced availability of UDP-GlcA further hampers HA production (Figure 2A) [56]. It is worth noting that the synthesis of other GAGs inside the Golgi is minimally affected by 4-MU, as transporters on the Golgi membranes ensure a high UDP-GlcA concentration in the organelle’s lumen.

It has also been reported that 4-MU can reduce the levels of mRNA coding for HASes [57] (Figure 2A). The mechanism behind this effect on gene expression remains unknown, but it could be an indirect consequence of an altered NAD:NADH ratio which, in turn, modulates sirtuins activity [58]. UDP-GlcA synthesis is catalyzed by UDP-glucose dehydrogenase (UGDH), which can doubly oxidase carbon 6 of UDP-glucose to form UDP-GlcA and generate two molecules of NADH (Figure 1A). Since 4-MU treatment reduces UDP-GlcA, one could speculate that compensatory mechanisms could lead to alterations in UDP-GlcA metabolism, resulting in an imbalance of the NAD:NADH ratio. Sirtuins, NAD^+^-dependent deacetylases, could therefore be deeply affected by 4-MU administration. Indeed, activation of sirtuin1 is known to reduce HA synthesis [59].

Given HA’s pivotal role in cancer biology, 4-MU treatment diminishes cancer cells´ aggressiveness in several tumor models, exerting potent antitumor effects primarily affecting HA-CD44 and HA-RHAMM downstream signaling pathways involving PI3K/Akt and MAPK/ERK, which are critical for cell survival, proliferation, and apoptosis. Interestingly, 4-MU has also been reported to enhance the efficacy of anticancer therapies, as described for carboplatin, trametinib, and cyclophosphamide [60,61,62,63]. HA size significantly impacts receptor activation and the activation of downstream signaling cascades. While HMW HA promotes CD44 clustering, HA oligomers diminish the clustering effect. This is because HMW HA has multiple binding sites for CD44, whereas oligomers have only one or two [64]. Therefore, the reduction of CD44 signaling can be achieved using very short HA oligosaccharides, which act as antagonists, decreasing the binding affinity of HMW HA and CD44, eventually limiting the receptor dimerization and inhibiting the signaling cascades (Figure 2B) [19].

It is noteworthy that even though 4-MU affects the metabolism of one of the main GAGs in our body, 4-MU treatment is generally well tolerated with minor side effects [65]. While 4-MU targets all three HASes by reducing UDP-GlcA levels, the effectiveness of inhibition on HAS1, HAS2, and HAS3 may vary. This variation arises from the different affinities these enzymes have for the substrates [66], which allows for basal HA synthetic activity.

**Figure 3:**
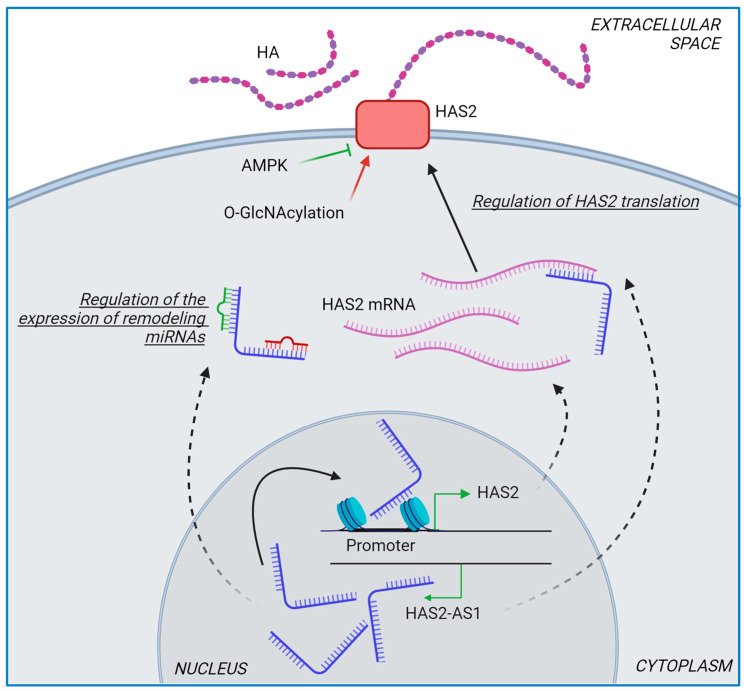
Schematic representation of the regulation of HAS2. Post-translational modifications by AMPK and O-GlcNAcylation modulate HAS2. HAS-AS1 can influence HAS2 in various ways, including altering chromatin structure at the HAS2 promoter, interacting with HAS2 mRNA, and serving as a sponge for miRNAs.

Among the three HASes, HAS2 plays a pivotal role in cancers and represents a potential target for reducing cancer aggressiveness [67]. Unlike HAS1 and HAS3, HAS2 undergoes post-translational modifications such as phosphorylation and O-GlcNAcylation. Phosphorylation of threonine-110, mediated by AMPK, inhibits HAS2 activity, while O-GlcNAcylation of serine-221 stabilizes HAS2 in the plasma membrane, thus enhancing HA production (Figure 3) [68].

Another unique aspect of HAS2 is the existence of the antisense transcript HAS2-AS1, or hyaluronan synthase 2 antisense RNA 1, located within the coding gene of HAS2 on chromosome 8 [69]. HAS2-AS1 is a long non-coding RNA (lncRNA) transcribed from the antisense strand of the HAS2 gene locus. Although HAS2-AS1 does not encode a protein, it regulates gene expression and cellular processes, with or without a direct link to its cognate gene HAS2, as reported elsewhere [69]. In non-small cell lung cancer, HAS2-AS1 accelerates gefitinib resistance by targeting the LSD1/EphB3 pathway [70], whereas in estrogen-negative breast cancer cells, HAS2-AS1 seems to have an antitumor activity independent of HA metabolism [67] (Figure 3).

### 4.2. Role of HYALs in Chemotherapy

The limited efficacy of chemotherapy in solid tumors can be partly attributed to the inefficient delivery of drugs within the altered TME. The TME, indeed, is characterized by an altered ECM, abnormal vasculature, reactive stroma, and persistent inflammation, which hinder the uptake, distribution, and efficacy of anti-neoplastic drugs. HA actively contributes to this process by forming a viscous gel that restricts the delivery, dispersion, and absorption of large volumes of drugs [23]. In this context, an alternative strategy involves the use of HYALs to degrade HA, thereby potentially enhancing tumor accessibility to anticancer drugs.

In recent years, PH-20 has emerged as a promising therapeutic agent for various cancer types. While numerous studies have explored the application of this enzyme and its commercial formulations, this section summarizes the most impactful advancements, particularly those involving the combination of PH-20 with conventional chemotherapy. ENHANZE^®^ technology is an approach to subcutaneous (SC) drug delivery based on a recombinant human PH20 (rHuPH20; Halozyme Therapeutics, Inc., San Diego, CA, USA), facilitating the co-administration of therapeutics and reducing patient burden compared to intravenous formulations [71].

rHuPH20, currently FDA-approved, is also co-formulated with two anticancer therapies, trastuzumab and rituximab. Trastuzumab is an antibody that targets human epidermal growth factor receptor 2 (HER2). Its combination with rHuPH20 is approved for SC administration for the treatment of HER2^+^ early- or metastatic breast cancer, and metastatic gastric cancer in the EU [72], for the treatment of HER2+ early- and metastatic breast cancer in Canada [73], and it is under review by the FDA for use in the US [74]. In addition, this combined strategy (Herceptin^®^, Roche Products Ltd., Mannheim, Germany) was used to generate an HA spherical nanocomplex for delivering HER2 blockade and paclitaxel (mitotic spindle inhibitor) chemotherapy. In vivo experiments have demonstrated significantly improved tumor growth inhibition compared to single-agent therapies with paclitaxel or Herceptin^®^ [75]. On the other hand, rituximab is an antibody that targets the CD20 protein present in pre-B- and mature B-lymphocytes. In combination with rHuPH20, rituximab is approved in the UE for its SC administration in the treatment of certain types of non-Hodgkin’s lymphoma [76]; in the US for chronic lymphocytic leukemia, follicular lymphoma, and diffuse large B-cell lymphoma [77]; and in Canada for non-Hodgkin’s lymphoma and chronic lymphocytic leukemia [78].

Afterward, PEGPH20, the PEGylated form of PH20, attracted significant research attention [79]. The attaching of a polyethylene glycol (PEG) molecule to PH20 extends its half-life and improves its efficacy compared to the unmodified form. Morosi et al. investigated the impact of PEGPH20 pre-treatment on paclitaxel in preclinical ovarian and pancreatic cancer models. They observed that pre-treatment with PEGPH20 altered tumor architecture, improved paclitaxel efficacy in the ovarian cancer model, and correlated with enhanced drug accumulation and more uniform intratumoral distribution. Additionally, PEGPH20 reduced HA content, influencing chemotherapy distribution and efficacy in the pancreatic model [80]. Moreover, a Phase Ib/II study investigated the combination of PEGPH20 with atezolizumab (anti-PD-L1 antibody) in advanced pancreatic and gastric cancer. Even though the safety profile of atezolizumab plus PEGPH20 was consistent, the combination showed limited clinical benefit [81]. Concurrently, a separate Phase III study compared atezolizumab delivery methods (SC vs. intravenous) in non-small-cell lung cancer. SC delivery of atezolizumab combined with PEGPH20 achieved a similar drug exposure, efficacy, safety, and immune response as intravenous, suggesting it as a viable alternative [82]. In addition to these studies, there are currently 22 active clinical trials investigating the combination of HYALs like PEGPH20 with various chemotherapies for a range of tumors, including B-cell and T-cell lymphomas, myeloma, endometrial, breast, lung, prostate, and gastric cancers, among others [83].

HYALs are currently under investigation for their potential to apply to oncolytic vaccinia viruses to enhance cancer therapy. These viruses are engineered to selectively replicate within tumor cells. One such example is VCN-01, an adenovirus designed to replicate in pancreatic cancer cells and express a HYAL. Studies have evaluated its antitumor efficacy, both alone and in combination with standard gemcitabine and nab-paclitaxel chemotherapy. The authors found that VCN-01 replicates within the tumor and reduces tumor stiffness, potentially facilitating the extravasation of chemotherapeutic drugs. This approach represents a promising new therapeutic strategy for cancers with dense stromal matrices, aiming to improve drug delivery and treatment efficacy [84].

Building upon the success of VCN-01, a similar strategy was developed for other cancer types, including breast and colon cancer. This approach utilizes a recombinant vaccinia virus, OVV-Hyal1, engineered to express a soluble form of HYAL1. Results demonstrated that OVV-Hyal1-mediated HA degradation facilitated the intratumoral distribution of chemotherapeutic drugs such as doxorubicin and gemcitabine. This enhanced drug delivery was accompanied by activation of the immune system, creating a synergistic antitumor effect. Notably, the combination of OVV-Hyal1 with chemotherapy yielded even more potent therapeutic outcomes [85].

In conclusion, HA catalysis and ECM remodeling due to HYALs´ activity represent a promising approach for enhancing drug delivery and improving cancer treatment outcomes. Ongoing clinical trials and investigations are evaluating the effectiveness and safety of HYAL–chemotherapy combinations across a range of cancer types, paving the way for potential breakthroughs in cancer therapy.

### 4.3. Role of UGDH in Chemotherapy

As outlined in the previous sections, HA is synthesized from UDP-GlcA and UDP-GlcNAc by HASes. Notably, UDP-GlcA serves as a versatile precursor in various biosynthetic pathways beyond HA synthesis. These pathways include the polymerization of other GAGs, such as HS and CS [58]. Additionally, this UDP-sugar significantly contributes to drug detoxification and clearance through glucuronidation reactions [86], serving as a protective mechanism that enhances the elimination of lipophilic xenobiotics from the body. Glucuronidation, involving drug conjugation to GlcA, is a pivotal metabolic pathway for several chemotherapeutic drugs, including steroid hormone analogs [87,88], the anthracycline epirubicin [89], and the topoisomerase inhibitors etoposide [90] and irinotecan [91], among other drugs widely used in the treatment of various types of cancer. UDP-GlcA is synthesized by the UGDH, which catalyzes a cytosolic NAD-dependent oxidation of UDP-glucose to produce UDP-GlcA [92,93] (Figure 1A). In the last decades, UGDH has emerged as a promising oncological target due to its relevance in glucuronidation detoxification and GAG synthesis [94]. Furthermore, UGDH has been correlated with the metabolism of tumor cells, owing to its role in the biosynthesis of sugar nucleotides. Specifically, a distinct linkage between UGDH and UDP-glucuronate decarboxylase 1 (UXS1) has been elucidated. UXS1 catalyzes the conversion of UDP-GlcA to UDP-xylose, and its indispensableness is evident exclusively in tumor cells with an upregulated UGDH expression (Figure 1A). This relationship arises as UXS1 is crucial for preventing the excessive accumulation of UDP-GlcA [95]. Consequently, these findings postulate UXS1 as a potential subject for future investigations exploring the interplay between UGDH and the metabolism of additional UDP-sugars in cancer.

As previously mentioned, androgen analogs undergo glucuronidation, a process dependent on UGT enzymes and UDP-GlcA availability. Notably, castration-resistant tumor cells, unresponsive to hormonal therapy, display elevated levels of UGDH and the UGT isoforms UGT2B15 and UGT2B17 [96]. Conversely, knocking down UGDH in tumor-derived xenografts not only diminished PGs but also suppressed androgen-dependent growth, regardless of androgen presence, and restored androgen sensitivity in castration-resistant cells. Importantly, UGDH knockdown in both androgen-dependent and castration-resistant cells dramatically sensitized them to enzalutamide, an androgen receptor antagonist [97]. These findings support the key role of UGDH in androgen responsiveness and remodeling of prostate tumor ECM, suggesting its potential as a therapeutic target for advanced prostate cancer [98].

Similarly, we investigated the role of UGDH in hormone-resistant breast cancer. TNBC lacks responsiveness to hormonal therapy, requiring treatment with conventional chemotherapy, such as anthracyclines as epirubicin [99], which is metabolized by glucuronidation. In this context, we observed a positive correlation between higher UGDH expression and poorer prognosis in TNBC patients who received chemotherapy. Furthermore, UGDH knockdown in epirubicin-treated MDA-MB-231 TNBC cells is associated with both epirubicin resistance and ECM remodeling. Notably, TNBC cells displayed increased epirubicin accumulation and decreased apoptosis, along with a positive modulation of autophagy, a mechanism previously proposed to promote epirubicin resistance [100]. Unexpectedly, a HA-coated matrix and HYAL expressions increased, suggesting a compensatory effect [101]. These findings support the proposal of UGDH as a novel prognostic marker in breast cancer, positively associated with the development of epirubicin resistance and modulation of the ECM. Our ongoing research explores the depletion of UDP-GlcA during epirubicin treatment using 4-MU [63]. Using two three-dimensional models of spheroids derived from breast cancer cells resistant and sensitive to hormonal therapy, we observed increased sensitivity to epirubicin treatment (unpublished results).

In recent years, the role of UGDH in drug resistance has also been investigated in other cancer types. Oyinlade et al. demonstrated a role for UGDH in glioblastoma, showing that UGDH knockdown in vitro and in vivo decreased GAGs accumulation and ECM proteins synthesis, along with diminished cell proliferation and migration [102]. Furthermore, a global proteomic profiling study of non-small cell lung carcinoma identified UGDH as a potential marker for etoposide chemoresistance. Etoposide and its analogs are widely used in lung cancer treatment, but the activation of drug resistance mechanisms is a significant challenge. The authors identified several potential chemoresistant markers in etoposide-resistant NCI-H460 cells using in silico analysis, highlighting UGDH and other candidates involved in chemoresistance. The expression of these candidates was further validated at the protein and mRNA level, opening new opportunities for investigating the molecular mechanisms underlying the development of etoposide resistance [103].

Another example of UGDH’s involvement in chemotherapy was observed in the treatment of hepatocellular carcinoma (HCC) with the kinase inhibitor sorafenib. It has been shown that sorafenib treatment in HCC cells activates UDP-GlcA metabolism and increases UGDH expression. Interestingly, an analysis of HCC patients receiving sorafenib treatment revealed that low UGDH expression predicted a better prognosis and a higher sorafenib efficacy [104]. In line with these findings, a recent study investigated the relationship between UGDH expression and the aggressive potential of ovarian cancers with distinct molecular profiles. The authors observed high UGDH expression specifically in high-grade serous ovarian cancers [105]. Notably, high UGDH expression correlated with a poor prognosis in the mesenchymal subtype, while low UGDH was associated with worse outcomes in the differentiated subtype. Furthermore, UGDH knockdown in the mesenchymal type led to diminished spheroid viability and decreased numbers of the CD133+/ALDHhigh population, a cell population known for its stem-cell-like properties and association with drug resistance [106,107].

Finally, several studies investigated the role of glucuronidation during drug resistance [108]. While not directly linked to UGDH expression, changes in UDP-GlcA bioavailability can indirectly influence glucuronidation pathways. For instance, a short communication on pancreatic cancer showed that blocking glucuronidation reactions significantly increased the effectiveness of gemcitabine, a nucleoside analog, in pancreatic ductal adenocarcinoma cells. Even more, UGT inactivation sensitized resistant tumor cells to gemcitabine treatment [109]. Similar results were obtained in studies on colon cancer, where glucuronidation was proposed as a mechanism of intrinsic drug resistance [110]. Specifically, the authors determined that glucuronidation is involved in the resistance to two topoisomerase I inhibitors used for colon cancer treatment. In agreement, the inhibition of glucuronidation increased drug activity. In addition, analysis of colon cancer biopsies revealed elevated expressions of UGT enzymes in most of the samples. These collective findings underscore the importance of further elucidating UGDH and glucuronidation as key mechanisms involved in cancer treatment and of exploring their therapeutic potential in future investigations.

### 4.4. Role of CD44-HA Interaction in Drug Resistance

One of the most important effects of HA on cancer cells is its modulation of signaling pathways through interaction with HA receptors, which regulate several intracellular kinase cascades [111,112]. By activating tyrosine kinase receptors, HA influences multiple pathways that significantly impact chemoresistance [15]. The main HA receptor is CD44, a cell-surface glycoprotein involved in cell–cell interactions, adhesion, and migration. CD44 plays a crucial role in various physiological processes, including lymphocyte activation, recirculation and homing, hematopoiesis, and tumor metastasis [113].

Structurally, CD44 includes an extracellular domain, a single-pass transmembrane domain, and a cytoplasmic tail. The HA-binding link module at the extracellular N-terminus mediates HA binding to CD44. The transmembrane domain anchors CD44 in the plasma membrane, while the intracellular domain mediates downstream signaling cascades by interacting with cytoskeletal proteins and signaling molecules, or by acting as a transcriptional factor [114].

CD44 exists in multiple isoforms generated by alternative splicing of the CD44 gene on chromosome 11. These isoforms can be broadly categorized into the standard (CD44s) and the variant forms (CD44v). CD44s is the shortest isoform, consisting of 10 constant exons without any of the variant exons. It includes the extracellular domain, transmembrane domain, and cytoplasmic tail and is involved in basic cellular functions such as cell adhesion, migration, and interaction with HA [115]. On the other hand, CD44v isoforms are generated by the inclusion of one or more variant exons (v1 to v10) in the extracellular domain, contributing to the structural and functional diversity of CD44, allowing for tissue-specific and context-specific functions, such as the ability to bind additional molecules other than HA (i.e., HS) and potential for post-translational modifications, including glycosylation [116]. CD44v isoforms are implicated in more specialized roles, particularly in cancer progression, metastasis, and chemoresistance. For example, CD44v3 and CD44v6 are often associated with enhanced cell migration, invasion, and metastasis, whereas CD44v8-10 is associated with the maintenance of CSC properties and chemoresistance [117,118,119].

CD44 downstream signaling pathways are generally conserved across various cell types and involve ErbB2, ezrin, PI3K-AKT, and MAP kinases, leading to the activation of survival mechanisms and antiapoptotic pathways [52]. HA-CD44 can also activate Rho signaling, which affects the cytoskeleton organization critical for mitosis, migration, and cisplatin resistance in head and neck cancer [120] (Figure 1B). Bourguignon et al. demonstrated that HA-CD44-mediated PKCϵ activation and subsequent Nanog phosphorylation and nuclear translocation results in miR-21 production, eventually promoting invasion and metastasis. Additionally, HA has been shown to enhance cell survival and reduce apoptosis induced by doxorubicin and paclitaxel, processes which are associated with an increase in inhibitors of apoptosis such as survivin and XIAP [121]. Other studies have demonstrated that HA reduces 5-fluorouracil sensitivity through TGFβ/Smad2-induced epithelial–mesenchymal transition in human gastric cancer cell lines [122].

HA-CD44 signaling also affects MDR by modulating the expression of ABC transporters. In breast cancer cells, HA is known to induce ABCB1 via CD44 signaling, reducing the efficacy of etoposide, doxorubicin, and paclitaxel [29,123]. In head and neck squamous cell carcinoma, the oncogenic protein deltaNp63 increases HA via HAS3 upregulation, and the HA-CD44 axis induces an ABCC1 (MRP1) transporter, conferring resistance to anti-neoplastic agents [124]. Similarly, other ABC members such as ABCC2, ABCB3, ABCC1, ABCC2, and ABCC3 are induced by HA signaling [28,125]. Moreover, high levels of HA interacting with CD44 and HER2 form a complex that finally activates Akt and induces ABCB1 gene expression, leading to doxorubicin resistance in colon and breast cancer cells [126].

As mentioned above, CSCs are considered a significant factor contributing to chemoresistance due to their specific characteristics, including a high expression of CD44. CD44 not only serves as a marker for CSCs but also plays an active role in regulating CSC properties due to its function as the main receptor for HA [127]. The enhanced HA-CD44 interaction plays a crucial role in maintaining the stemness properties of CSCs by activating signaling pathways like Wnt/β-catenin and Hedgehog. Moreover, as reported above, ABC transporters are upregulated to increase drug resistance, and antioxidant pathways mediated by NRF2 are induced by HA-CD44, protecting CSCs from ROS-induced apoptosis [128]. Additionally, the binding of CD44 to HA can activate pathways involved in epithelial–mesenchymal transition, allowing CSCs to become more motile and invasive [129]. In an isolated CSC-like population from human head and neck squamous carcinoma, HA-activated survival proteins confer chemoresistance to cisplatin [130].

## 5. Future Perspectives and Conclusions

Chemotherapy resistance represents a major cause of therapeutic failure that leads to mortality in cancer patients. The complexity of tumor ECM, particularly involving cancer cells and HA, presents both challenges and opportunities for developing effective cancer therapies. Targeting the HA-ECM network offers a promising avenue for improving cancer therapy. By disrupting HA-mediated physical barriers and signaling pathways, we can enhance the delivery and efficacy of several chemotherapeutic agents. However, the promise of HA-based cancer therapy is tempered by the intricate interplay between HA synthesis and degradation mechanisms, representing a challenge. The dynamic regulation of HA polymer lengths within the TME adds another layer of complexity to controlling HA’s therapeutic potential. Future research should address these challenges to better understand the know-how of the clinical application of HA-based therapy. Additionally, targeting therapies that include HYALs, HA-CD44 interaction inhibitors, and epigenetic modulators represents a potential multi-faceted approach to overcome chemoresistance and improve outcomes for patients with HA-rich tumors.

Another essential aspect to consider when screening for new anticancer treatments is selecting the most appropriate and reliable experimental model. Even if two-dimensional cell culture remains the gold standard method to evaluate the efficacy of new drugs in preclinical studies, this kind of culture does not provide a comprehensive understanding of cell–cell and cell–ECM interactions occurring in real tumors. To better reproduce the structural organization of actual solid tumors, three-dimensional cultures (mainly spheroids) have been developed during the last few decades. HA plays a major role in this setting, as it can be used to produce three-dimensional structures, helping spheroids self-assemble. Moreover, being highly involved in tumorigenesis, tumor aggressiveness, and, as discussed in this review, chemoresistance, HA inclusion in three-dimensional cultures could provide a better insight into interactions and signaling pathways occurring in vivo between cancer cells, HA, and the TME in general.

## Data Availability

Figures presented in this review were created with BioRender.com, accessed on 30 May and 3 June 2024, with agreement numbers AF26VQBAZ2, YT26VQBEPZ, MH26VQC8CU, and GX26W9LH4F to A.P. (Arianna Parnigoni).
